# Bursting suppression in propofol-induced general anesthesia as bi-stability in a non-linear neural mass model

**DOI:** 10.1186/1471-2202-15-S1-P139

**Published:** 2014-07-21

**Authors:** Pedro Garcia-Rodriguez, Axel Hutt

**Affiliations:** 1 INRIA CR Nancy-Grand Est, Equipe NeuroSys, France

## 

**Figure 1:**
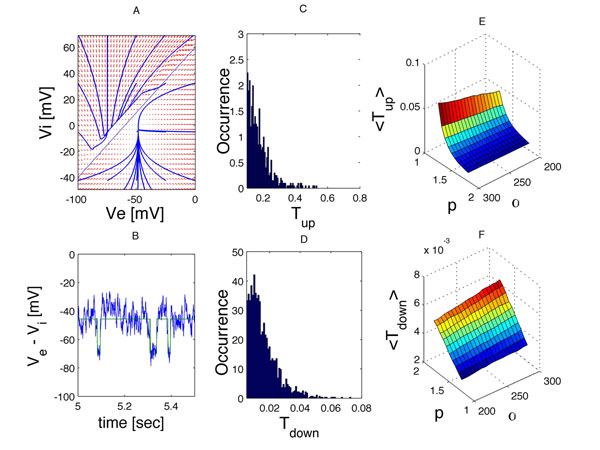
Bi-stability in a simplified neural-mass version of the Hutt & Longtin model [3], with neuron firing rates functions of Heaviside kind. **A.** 2D phase-portrait in the PSPs membrane potentials, showing the vector field (arrows) and sample free-noise trajectories (blue) that converge to either of two existing attractors. An oblique line, containing unstable (saddles) states, largely separates the attraction basins. **B.** Noise-induced alternations between a larger (*up* state) and a smaller (*down* state) value can be tracked according to the current distance of the trajectory to the saddle line. **C**, **D.** Histograms of the residence times in each of the stable states. **E, F**. The mean residence times as a function of the propofol level (*p*) and the inverse of the excitatory synaptic impulse response time-scale (*α*).

Bursting activity suppression, a phenomenon characterized by sequences of alternating quiescent (’*down*’) and bursting (’*up*’) states, is ubiquitously present during deep sedation in general anesthesia and can be observed in LFPs and in EEG recordings as well [1,2]. However, the dynamical principles underlying such phenomena are unclear. Prevailing theoretical approaches to this problem suggest that such bi-stable dynamics could be explained by the existence of notable non-linearities in the neuronal membrane potential as a function of the anesthetic agent, with frequent noise-induced transitions between two stable (attracting) branches. Nevertheless, the mathematical tractability of those models is rather limited due to the dimensionality (2D) of the phase-space (see Figure 1). It has been shown for a neural-mass version of the model proposed in [3], that the 2D-trajectory likely escapes to the other attractor trough a limited section of the saddle-line separating the attractions basins [4]. This prediction is here supported by numerical simulations, permitting the simplification of the dynamics to a simpler two-state trace. Relevant statistics can then be extracted, including histograms of the duration of the visit in each state that may support future analytical calculations of the EEG power spectrum following renewal theory [5]. Interestingly, it is found that only the average waiting time in the '*up*' states is a non-linear decreasing function of *p*, differently to the residence time in the ’*down*’ state that behaves linearly in *p* and in the synaptic time-scale (1/*α*) direction as well.
